# Low-temperature synthesis of di- and monoolein by enzymatic hydrolysis in a biphasic system using cutinase from *Fusarium graminearum*

**DOI:** 10.1007/s10068-025-02062-z

**Published:** 2026-01-24

**Authors:** Juchan Lee, Juno Lee, Jihoon Kim, Pahn-Shick Chang

**Affiliations:** 1 Department of Agricultural Biotechnology, Seoul National University, Seoul, 08826 Republic of Korea; 2 Research Institute of Agriculture and Life Sciences, Seoul National University, Seoul, 08826 Republic of Korea; 3 Center for Agricultural Microorganism and Enzyme, Seoul National University, Seoul, 08826 Republic of Korea; 4 Center for Food and Bioconvergence, Seoul National University, Seoul, 08826 Republic of Korea

**Keywords:** Functional lipid, Oleoylglycerol, Lipase, Low temperature, *Fusarium solani* cutinase

## Abstract

Among functional lipids, oleoylglycerols including 1,2-diolein and 2-monoolein are known for their beneficial activities such as fatty acid metabolism, insulin secretion promotion, antioxidant, and anti-arrhythmic sclerosing activity. To produce these oleoylglycerols through the lipase reaction, cutinase from *Fusarium graminearum* (FGC) were recombinantly expressed and the properties of the enzyme were analyzed. The enzyme exhibited optimal activity under weak alkaline conditions (pH 8–9) and low temperatures (2–10 °C) for triolein in the water–isooctane biphasic system. The product of the lipase reaction with FGC at a low temperature was analyzed by HPLC, revealing that FGC appeared as *sn*-1(3) regiospecific lipase and only 2-monoolein was produced in monoolein isomers with 15.7 ± 3.83% conversion at 15% hydrolysis of triolein. These results showed that the FGC can be used to produce 1,2-diolein and 2-monoolein and further suggest its potential as a versatile enzyme in lipid modification in low-temperature environments.

**Supplementary Information:**

The online version contains supplementary material available at 10.1007/s10068-025-02062-z.

## Introduction

Functional lipids are lipids with various physiological functions such as antioxidants, hormone control, and immune control as well as the role of energy storage and supply. Examples of functional lipids include steroids, carotenoids, and phosphatidylcholine (Omachi et al., 2024). Triacylglycerol, the most common form of lipid, is generally considered a nutritional energy source, but when composed of special fatty acids such as omega-3 and omega-6 fatty acids, it acts as a functional lipid to improve cardiovascular health and control inflammation (Calder, 2015). Another type of functional lipid in the form of triacylglycerol is structured lipids, which have a modified fatty acid composition. For example, a structured triacylglycerol with a medium- and long-chain fatty acid composition is expected to improve absorption rates and promote metabolism in the body (Wang et al., 2022).

Monoacylglycerols and diacylglycerols are acylglycerol lipids that are produced by the partial hydrolysis of triacylglycerols. Due to their excellent surfactant properties, they have been used as emulsifiers in the food industry (Krog, 1977). These lipids are also used as functional lipids that exhibit pharmacological activity in vivo. For example, diacylglycerol influences fatty acid metabolism and has physiological effects related to regulating body fat accumulation (Nagao et al., 2000). Certain monoacylglycerols, such as 2-monoolein, stimulate insulin secretion via GPR119 receptors (Hansen et al., 2011). Additionally, 2-monoolein is attracting attention as a functional lipid due to its excellent antioxidant and anti-arrhythmic sclerosing activity, as demonstrated in vitro and in cell-level studies (Cho et al., 2010). These characteristics endow di- and monoolein with high industrial value for broad applicability in food, pharmaceuticals, and medical fields.

Although chemical methods can be used to prepare functional lipids, there are disadvantages such as harsh reaction conditions and complications in the purification due to byproducts. Accordingly, eco-friendly and highly selective enzyme-based synthesis strategies are attracting more attention (Guan et al., 2024). For example, transesterification and acidolysis reactions using lipase are widely used to manufacture structured lipids (Akil et al., 2020; Utama et al., 2019). When preparing functional lipids containing omega-3 fatty acids, lipase is used for the alcoholysis reaction to obtain monoacylglycerol and for the esterification reaction to produce recombinant triglycerides (Haq et al., 2020; Solaesa et al., 2016; Xia et al., 2019). Diaclyglycerols and monoacylglycerols are synthesized through lipase reactions, such as the partial ethanolysis of triacylglycerols or the acylation of glycerol. In particular, for producing position-specific acylglycerols (e.g., 2-monoolein), regioselective lipases such as *Thermomyces lanuginosus* lipase and *Rhizomucor miehei* lipase can be utilized (Abreu Silveira et al., 2017; Whitten et al., 2012).

In the enzymatic synthesis of functional lipids, both enzyme and lipid substrates can be denatured by high temperatures. When lipids react at high temperatures, there are problems such as the spontaneous oxidation by oxygen, hydrolysis due to reaction with moisture, and rapid degradation of the quality of lipids (Choe & Min, 2006). This is more sensitive to fatty acids with higher levels of unsaturation. Therefore, it is advantageous in terms of substrate stability to perform the lipid reaction at low temperatures. To perform efficient reactions under these conditions, cold-adaptive lipases with high activity at low temperatures can be used. They are primarily derived from the enzymes of polar microorganisms and exhibit high lipase activity at low temperatures (Feller and Gerday, 2003). However, most cold-adaptive lipase studies focused on temperature characteristics, while investigation on the lipase reaction characteristics, such as regioselectivity, remains insufficient.

Plant-pathogenic fungi, such as *Fusarium*, are a food safety concern due to their production of toxic substances, such as mycotoxins (Bondoc, 2016). However, the hydrolytic enzymes that these fungi secrete to degrade plant cell walls, including lipases and cutinases, are of considerable interest to the food industry. Among them, *Fusarium solani* cutinase has been studied for a long time, and its structure and activity properties are well identified (Egmond and de Vlieg, 2000). This enzyme was originally identified as an esterase that decomposes plant cutin polymers. However, it also exhibits lipase properties and degrades polymer esters, such as polyethylene terephthalate (Ronkvist et al., 2009). The enzyme has been reported to exhibit *sn*-1(3) regioselective lipase activity (Rogalska et al., 1993). There are many other *Fusarium* species that also have cutinases; however, most of these enzymes have not yet been studied. Given their potential as a source of novel lipase candidates, it is crucial to investigate the lipase characteristics of cutinases in *Fusarium*.

In this study, cutinase from *Fusarium graminearum* (FGC), which has a homologous sequence with *F. solani* cutinase, was expressed and the hydrolysis characteristics of the enzyme were analyzed. FGC selectively produced 1,2(2,3)-diolein and 2-monoolein in triolein hydrolysis at low temperatures in a water-isooctane biphasic system. The results of this study show that FGC can be used for the biosynthesis of functional lipids and suggest its potential as a useful enzyme in expanding into various functional lipid production platforms in low-temperature environments, which makes it valuable in lipid industries.

## Materials and methods

### Chemicals, plasmids, and strains

The expression host *Pichia pastoris* X-33 and vector pPICZα A were purchased from Invitrogen (Thermo Fisher Scientific Inc., Waltham, MA, USA). Zeocin was purchased from InvivoGen, Inc. (San Diego, CA, USA). *F. graminearum* was provided by Korean Agricultural Culture Collection (KACC; strain no. 41040). *Escherichia coli* DH5α was purchased from Dyne Bio Inc. (Gyeonggi, Republic of Korea).

Pierce Bradford Plus Protein Assay Reagent from Thermo Fisher Scientific was used. Triolein (≥ 99%), *p*-nitrophenyl palmitate (*p*-NPP; ≥ 98%), sodium dodecyl sulfate (SDS), Triton X-100, and Tris were purchased from Sigma-Aldrich, Inc. (St. Louis, MO, USA). Sodium acetate and sodium chloride were purchased from Junsei Chemical Co. Ltd. (Tokyo, Japan) and acetic acid, acetonitrile (HPLC grade), and acetone (HPLC grade) were purchased from Samchun. Yeast nitrogen base without amino acid and ammonium sulfate was purchased from Kisanbio Co. Ltd. (Seoul, Republic of Korea), methanol was purchased from Samchun Pure Chemical Co. Ltd. (Gyeonggi, Republic of Korea), yeast extract and glucose were purchased from Thermo Fisher, peptone, sorbitol, and ammonium sulfate were purchased from Duksan Pure Chemicals Co. Ltd. (Gyeonggi, Republic of Korea). All other chemicals were used in reagent grade.

## Expression and purification of FGC

### Construction of expression strain

Protein expression was performed according to the protocol of Weidner et al. (2010). The cutinase transcript sequence (NCBI reference sequence: XP_011322914.1) homologous to the *F. solani* cutinase protein sequence was reverse transcribed from the *F. graminearum* and amplified excluding sequences according to the pre- and pro-regions according to van Gemeren et al. (1995). RNA-spin Total RNA Extraction Kit (Intron biotechnology Inc., Gyeonggi, Republic of Korea), and SuperScript III First-Strand Synthesis System (Thermo Fisher) were used for RNA extraction and cDNA construction. AccuPower HotStart Pfu PCR PreMix, AccuPrep PCR/Gel Purification Kit and AccuPrep Plasmid Mini Extraction Kit from Bioneer (Daejeon, Republic of Korea) were used for extraction and purification of amplified DNAs. The amplified sequence was cloned downstream with AccuRapid Cloning Kit (Bioneer) of the Kex2 cleavage site in the pPICZα A vector with *Xho* I restriction enzyme (New England Biolabs, Ipswich, MA, USA) to express the protein with a native N-terminus. The amplified sequence contained a stop codon for the expression of the protein with a native C-terminus. The constructed expression vector was extracted, linearized by *Pme* I (New England Biolabs), and transformed into competent *P. pastoris* cell by electroporation with electroporation cuvettes and electroporator (Gene Pulser/MicroPulser Electroporation Cuvettes, 0.2 cm gap and MicroPulser Electroporation Apparatus) from Bio-Rad (Hercules, CA, USA). Zeocin was used to select the pPICZα A vector insertion at concentrations of 50 µg/mL for *E. coli* and 200 µg/mL for *Pichia*.

## Protein expression

Yeast nitrogen base (YNB) was prepared at a tenfold concentration by using 0.34% (w/v) yeast nitrogen base without amino acids and without ammonium sulfate with 1% (w/v) ammonium sulfate. Buffered glycerol-complex medium (BMGY; 1% (w/v) yeast extract, 2% (w/v) peptone, 1% (w/v) glucose, 1% (w/v) YNB, and 1% (v/v) glycerol with 100 mM sodium phosphate buffer at pH 6.0) and buffered methanol-complex medium (BMMY; 1% (w/v) yeast extract, 2% (w/v) peptone, 1% (w/v) glucose, 1% (w/v) YNB, and 0.5% (v/v) methanol with 100 mM sodium phosphate buffer at pH 6.0) were used for incubation and induction of *Pichia* expression strain. The strain was pre-incubated at 28 °C in BMGY medium and then transferred to BMMY expression medium to an optical density of 1.0 at 600 nm. Expression strains were incubated for three days at 25 °C to produce recombinant proteins, with 0.5% (v/v) methanol added daily.

## Protein purification

The expression media were centrifuged at 3,000 × *g* and then filtered through a 0.45 µm regenerated cellulose filter to remove cell residues. Protein concentration and buffer change were performed by ultrafiltration with the protein concentrator (10 kDa molecular weight cutoff, Satorius AG, Göttingen, Germany) Protein purification was performed using cation exchange chromatography with an ÄKTA go FPLC system (Cytiva, Marlborough, MA, USA). HiTrap CM FF 5 mL column from Cytiva was used as the column for ion exchange chromatography. The filtered media were concentrated, changed with the start buffer (50 mM sodium acetate buffer, pH 5.0) and then injected into the column. Proteins were eluted by stepwise increasing the concentration of the elution buffer (50 mM sodium acetate with 1 M NaCl, pH 5.0). The eluted and concentrated fractions were changed with 50 mM Tris–HCl (pH 8.0) buffer. Bradford assay and lipase assay for *p*-NPP were used for activity evaluation. SDS-PAGE was performed on samples before and after the ion exchange chromatography purification to check the size of the expressed protein.

## Lipase assay

### *p*-NPP assay

To determine *p*-NPP hydrolysis activity, a substrate solution consisting of 2 mM *p*-NPP, 1% (w/v) Triton X-100, and 0.588 mM SDS was prepared by heating to 60 °C. The enzyme reaction was initiated by adding an equal volume of the substrate solution to the buffered enzyme solutions. For pH variation, a 120 mM Britton–Robinson buffer was used, and for temperature variation, a 50 mM Tris–HCl (pH 9.0) buffer was used. The assay for pH variation was performed in a 96-well microplate with a total volume of 200 µL, with 3 s of shaking at 1 min intervals. For temperature variation, it was performed in a vial with a total volume of 2 mL with magnetic stirring at 700 rpm. Concentration of liberated *p*-nitrophenol was measured at 347 nm using a SpectraMax iD3 microplate reader (Molecular Devices, San Jose, CA, USA). The effects of temperature and pH on lipase activity were evaluated at various temperatures (10–60 °C) and pH values (4–12).

## Triolein hydrolysis in a biphasic system

The method described by Lee et al. (2024) was slightly modified for the lipase reactions in a water–isooctane biphasic system. A 40 mM solution of triolein in isooctane was used as a substrate. The enzyme solution was prepared in an appropriate buffer solution (50 mM sodium phosphate buffer for pH 6–7, 50 mM Tris–HCl buffer for pH 7–9, or 50 mM sodium carbonate buffer for pH 9–10). The reaction was carried out in a water bath with magnetic stirring at 700 rpm and initiated by adding an equal volume of the substrate solution to the 2 mL of enzyme solution. The concentration of liberated oleic acid in the sampled organic phase was measured using a colorimetric method with a 5% (w/v) cupric acetate–pyridine solution (pH 6.1) as the colorant. The absorbance of the organic phase at 715 nm was measured after the reaction by mixing the colorant and the organic phase at a volumetric ratio of 1:5. The effects of temperature and pH on lipase activity were evaluated at various temperatures (2–40 °C) and pH values (6–10).

## HPLC-ELSD analysis

The analysis method described by Choi et al. (2022) was used to evaluate the degree of hydrolysis and monoolein production in a water–isooctane biphasic system. The organic phase was sampled at various time points and dissolved in acetonitrile/acetone (90:10, v/v). An HPLC separation was carried out using a CHIRALPAK IA column (Daicel Chemical Ind., Osaka, Japan) equipped with a Waters Alliance e2695 HPLC separation module (Waters Corp., Milford, MA, USA) and an evaporative light scattering detector (ELSD) system (Alltech ELSD 2000 system, Alltech Associates Inc., Deerfield, IL, USA). The ELSD drift tube and nebulizer temperatures and the nitrogen gas flow were 70 °C and 1.8 L/min, respectively. The mobile phase was acetonitrile/acetone/trifluoroacetic acid (90:10:0.1, v/v/v) and the flow rate was 0.5 mL/min.

## Statistical analysis

All lipase assays were performed at least three times and noted as mean with standard error. Statistical significance was verified using analysis of variance (ANOVA) and the Tukey post-hoc test in IBM SPSS Statistics 29 software (SPSS Inc., Chicago, IL, USA) with significance level *p* < 0.05.

## Results and discussion

### Heterologous expression of FGC

When proteins are expressed recombinantly, they can be easily and highly purified by expressing them with a tag for purification (Young et al., 2012). In the case of *F. solani* cutinase the orientation of the C-term is opposite to the catalytic site (Egmond and de Vlieg, 2000). Thus, introducing a C-term tag may be an effective method without significantly affecting the enzyme properties. However, Kwon et al. (2009) reported that the introduction of the C-term tag reduced expression levels and adversely affected protein folding. To minimize the effect on the properties of the enzyme, the enzyme was expressed in its native form and purified by ion exchange chromatography.

**Fig. 1:**
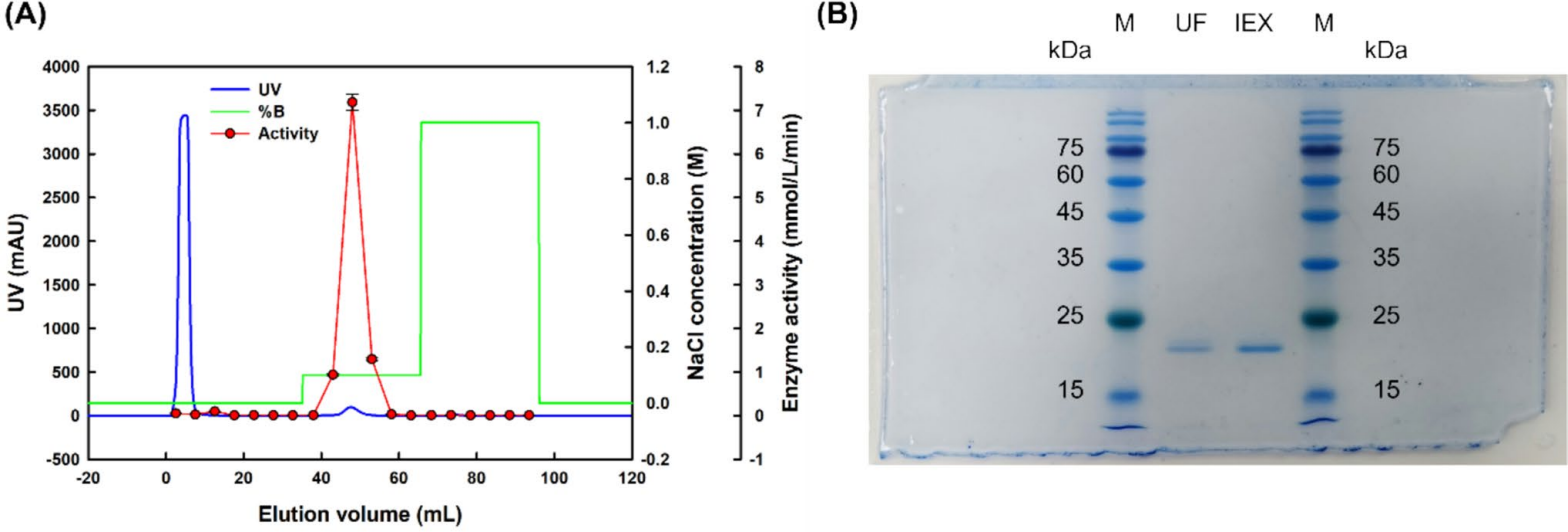
Cation exchange chromatography and SDS-PAGE results of FGC. (**A**) Cation exchange chromatography results, (**B**) SDS-PAGE results. M: protein marker, UF: crude media after ultrafiltration, IEX: sample after ion exchange chromatography. Enzyme elution: 0–0.1 M NaCl concentration

The expressed protein was eluted in fractions at a concentration of 0–0.1 M NaCl. Changes in activity before and after purification are shown in Table 1. After the ion exchange chromatography, the specific activity of the lipase of the sample increased. The results of the SDS-PAGE analysis for the FGC sample after ultrafiltration and for the sample after purification with ion exchange chromatography are shown in Fig. 1. The protein band became clearer after purification and appeared at around 20 kDa, which is the expected size of the FGC protein based on its amino acid sequence. The expression and purification of the recombinant protein were performed well with an active form of lipase.

**Table 1:** Purification table of FGC

Step	Total protein (mg)	Total activity (U^*^)	Specific activity (U^*^/mg)	Purification (fold)	Yield (%)
Ultrafiltration	10.85 ± 0.16	752 ± 37	69.3 ± 3.2	1	100
IEX (CM)	6.95 ± 0.28	634 ± 38	91.3 ± 4.1	1.32 ± 0.09	84.4 ± 7.7

^*^One unit (U) is defined as the amount of enzyme that releases 1 µmol of product per minute in the *p*-NPP assay at pH 8.0 and 40 °C

## Determination of the optimal hydrolysis reaction conditions for FGC

**Fig. 2:**
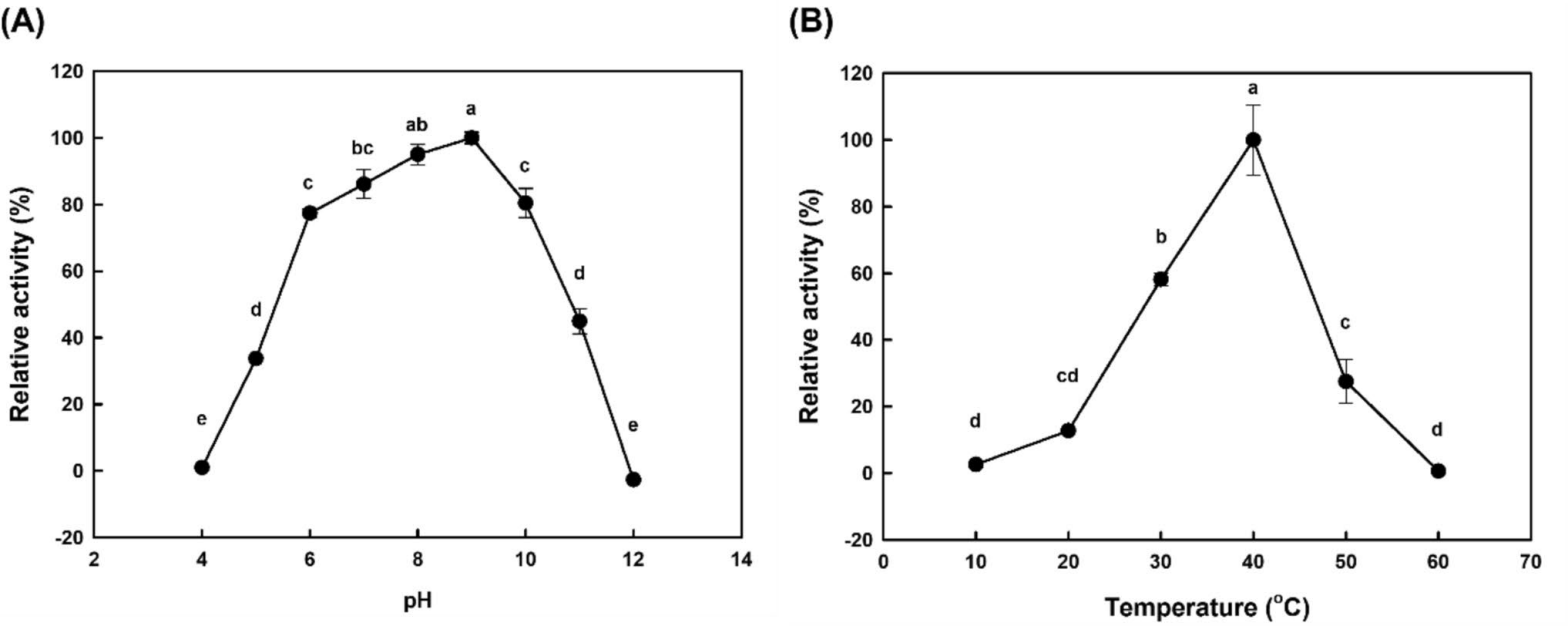
Lipase assay results for *p*-NPP of FGC by pH and temperature variation. (**A**) pH variation, (**B**) temperature variation. The pH variation experiments were conducted at a reaction temperature of 40 °C using 120 mM Britton–Robinson buffers at different pH values. For temperature variation experiments, 50 mM Tris–HCl buffer at pH 9.0 were used. The relative activities at 100% were as follows: 79.2 U/mg for pH variation and 14.7 U/mg for temperature variation

**Fig. 3:**
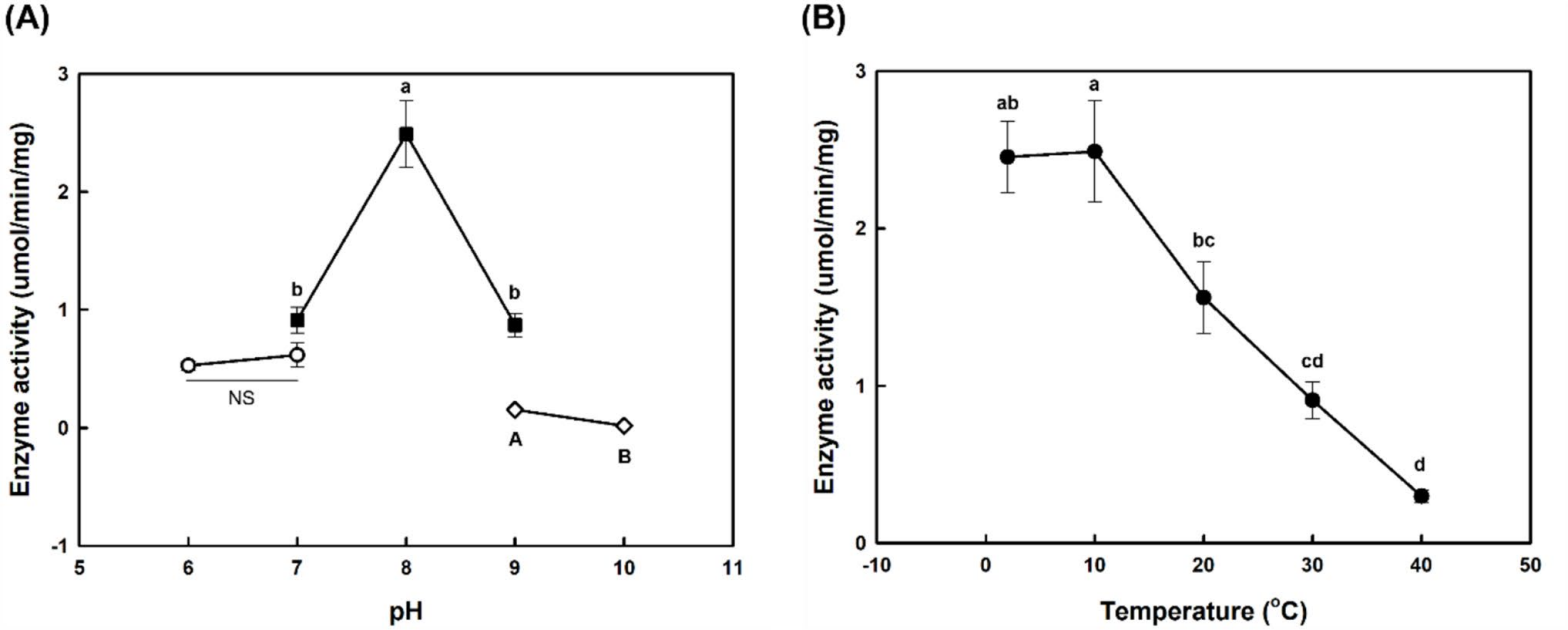
Lipase assay results for triolein of FGC in water–isooctane biphasic system by pH and temperature variation. (**A**) pH variation, (**B**) temperature variation. As a substrate solution, 40 mM of triolein in isooctane was used and 50 mM sodium phosphate (white circles), Tris–HCl (black squares), sodium carbonate (white diamonds) buffer and 50 mM Tris–HCl (pH 8.0) buffer (black circles) were used as an enzyme solution. NS: not significant. The highest activity: 2.49 U/mg

Figure 2 shows the comparison of *p*-NPP hydrolysis activity with variations in pH and temperature. Heat inactivation of the enzyme was shown by a decrease in activity over time during 15 min reactions at temperatures of 50 °C or higher (Fig. S1). The optimal conditions for enzyme activity were pH 8–9 and a temperature of 40 °C. The comparison of triolein hydrolysis activity in a water–isooctane biphasic system with variations in pH and temperature is shown in Fig. 3. The optimal conditions for enzyme activity were pH 8 and a temperature of 2–10 °C.

The low stability of *F. solani* cutinase in organic solvents can be attributed to its low activity at high temperatures (Melo et al., 1995). However, for FGC, the residual activity of the enzyme did not significantly decrease during the initial 2 h, and there was no significant difference according to temperature between 10 °C and 40 °C in a water–isooctane biphasic system (Fig. S2). These results indicate that the decrease in activity at higher temperatures is unlikely due to enzyme denaturation by heat or organic solvent. Further investigation with *p*-NPP in a water–isooctane biphasic system showed optimum temperature shift to lower temperatures (Fig. S3), demonstrating that this enzyme FGC exhibits low-temperature activity in a biphasic system.

Unlike general lipases, cutinase does not have a lid structure that opens at the interface (Egmond and de Vlieg, 2000). For this reason, the enzyme does not exhibit interfacial activation in which lipase activity appears only when the substrate concentration exceeds the critical micelle concentration (Longhi and Cambillau, 1999). As the catalytic site of the enzyme is constantly exposed to the solvent, the enzyme can be affected by the surrounding solvent environment. The low-temperature activity of FGC in a water–isooctane biphasic system is probably due to the open catalytic structure and the reaction environment.

## Production of di- and monoolein by FGC

**Fig. 4:**
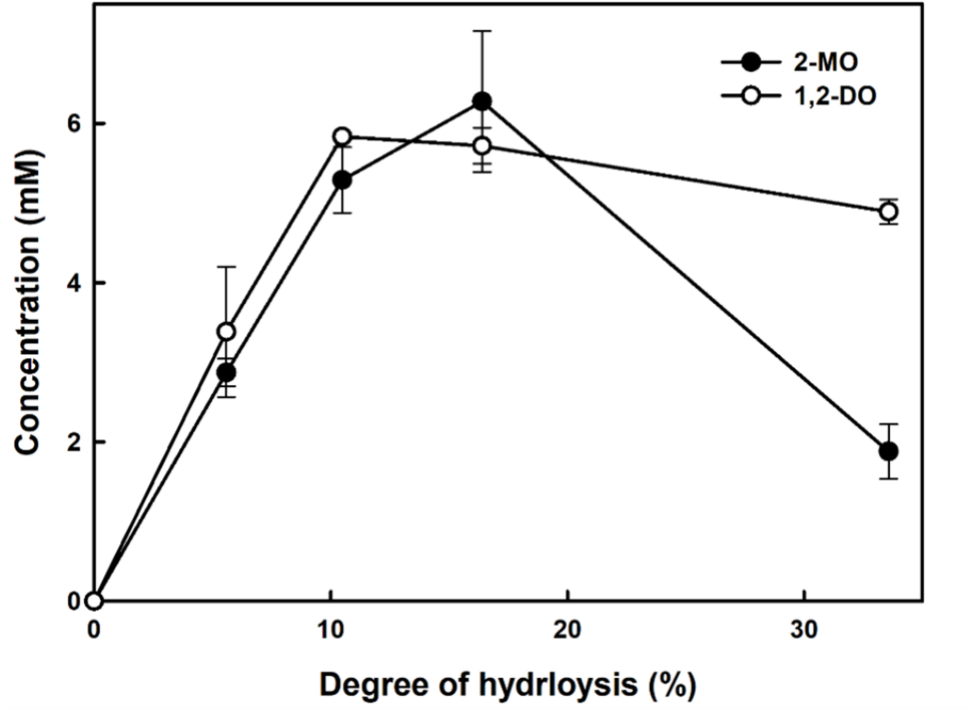
Concentration of 1,2(2,3)-diolein and 2-monoolein in lipase assay with FGC in water–isooctane biphasic system. Triolein (40 mM) in isooctane was used as a substrate and enzyme with 50 mM Tris–HCl (pH 8.0) buffer was used. Copper soap colorimetric assay was used for fatty acid quantification. 2-MO (black circles): 2-monoolein, 1,2-DO (white circles): 1,2(2,3)-diolein

An *sn*-1(3) regiospecific lipase, *Rhizopus oryzae* lipase, was previously reported in the production of 2-monoolein. Mangas-Sánchez (2015) found that up to 76% of the monoolein was produced using the ethanolysis method with *n*-heptane and ethanol. Ghattas (2014) produced 78% monoolein in a 10% (w/v) gum Arabic emulsion system. Both studies conducted reactions at 37 °C, which is the optimal temperature for the enzyme. In this study, 1,2(2,3)-diolein and 2-monoolein were produced using FGC by performing the triolein hydrolysis reaction in a water–isooctane biphasic system. The triolein hydrolysis reaction was performed at a temperature of 10 °C, taking advantage of the low-temperature activity characteristics of the FGC in a water–isooctane biphasic system. Since the optimal hydrolysis reaction temperature conditions for FGC differed from those reported for *F. solani* cutinase, diolein was used to confirm that the regioselectivity of lipase did not differ. It was found that 1,3-diolein was below the detection limit (Fig. S4). This indicates that FGC is an *sn*-1(3) specific lipase, like *F. solani* cutinase. Throughout the reaction, 2-monoolein was the only observed monoolein. Meanwhile, Fig. 4 shows the 2-monoolein levels according to the degree of hydrolysis. As the hydrolysis reaction progressed, the concentration of 2-monoolein increased to a maximum concentration of 6.28 ± 0.89 mM at 16.39 ± 1.15% degree of hydrolysis and then decreased to 1.88 ± 0.34 mM at 33.57 ± 0.64% degree of hydrolysis. This indicates a difference in hydrolysis selectivity between triolein and monoolein.

Most studies of lipases, including *F. solani* cutinase, investigate the ratio of diacylglycerol produced through the hydrolysis of triacylglycerol for the evaluation of regioselectivity (Rogalska et al., 1993, 1990). According to this method, FGC is an *sn*-1(3) specific lipase because it does not produce 1,3-diolein when it hydrolyzes triolein. However, this method does not reveal lipase selectivity for diacylglycerol and monoacylglycerol. Based on the analysis of the entire triacylglycerol hydrolysates in the form of acylglycerol, Choi et al. (2022) proposed the concept of integral lipase stereoselectivity, which can present lipase positional selectivity for diacylglycerol and monoacylglycerol as dynamic constants. Since the 2-monolein is hydrolyzed in the triolein hydrolysis reaction with FGC, introducing the concept of integral stereoselectivity will help to increase the understanding of the selectivity of FGC and use the enzyme as a regioselective lipase for lipid modification.

In this study, an enzyme with lipase activity homologous to the *F. solani* cutinase which exhibits *sn*-1(3) lipase activity was discovered in *F. graminearum* and the recombinant protein was expressed in *Pichia* system. The enzyme exhibited hydrolysis activity against triolein in a water–isooctane biphasic system at low temperatures, selectively producing 1,2(2,3)-diolein in diolein isomers and 2-monoolein in monoolein isomers. Biphasic systems offer several advantages for lipase-catalyzed reactions, including separating the enzyme and products, improving substrate solubility, and enabling continuous monitoring of the reaction. A novel enzyme found in this study, FGC, exhibits high activity at low temperatures in a biphasic system and is expected to be used to establish a production process for functional lipids in a low-temperature environment. The reaction under low-temperature conditions can suppress lipid oxidation and quality deterioration, providing great advantages in ensuring product stability in functional lipid production. Further investigation of the regioselectivity of the lipase can increase the utilization value of the enzyme.

## Supplementary Information

Below is the link to the electronic supplementary material.

Supplementary file1 (DOCX 258 KB)

## Data Availability

Data will be made available on request.
