# Supplementary material for: Low-temperature synthesis of di- and monoolein by enzymatic hydrolysis in a biphasic system using cutinase from Fusarium graminearum
Source: Food Sci Biotechnol. 2026 Jan 24;35(3):493–500. doi: 10.1007/s10068-025-02062-z (PMC12894595; doi:10.1007/s10068-025-02062-z)
Supplement: Supplementary file 1 — Supplementary file1 (DOCX 258 KB) [file 10068_2025_2062_MOESM1_ESM.docx]

**Supplementary Figures**

**Low-temperature synthesis of 2-monoolein by enzymatic hydrolysis in a biphasic system using cutinase from *Fusarium graminearum***

Juchan Lee^a,1^, Juno Lee ^b^, Jihoon Kim^a^, Pahn-Shick Chang^a,b,c,d*^

^a^ *Department of Agricultural Biotechnology, Seoul National University, Seoul 08826, Republic of Korea*

^b^ *Research Institute of Agriculture and Life Sciences, Seoul National University, Seoul 08826, Republic of Korea*

^c^ *Center for Agricultural Microorganism and Enzyme, Seoul National University, Seoul 08826,*

*Republic of Korea*

^d^ *Center for Food and Bioconvergence, Seoul National University, Seoul 08826, Republic of Korea*

* Author to whom correspondence should be addressed [telephone: +82 2 880 4852; fax: +82 2 873 5095; e-mail: pschang@snu.ac.kr]


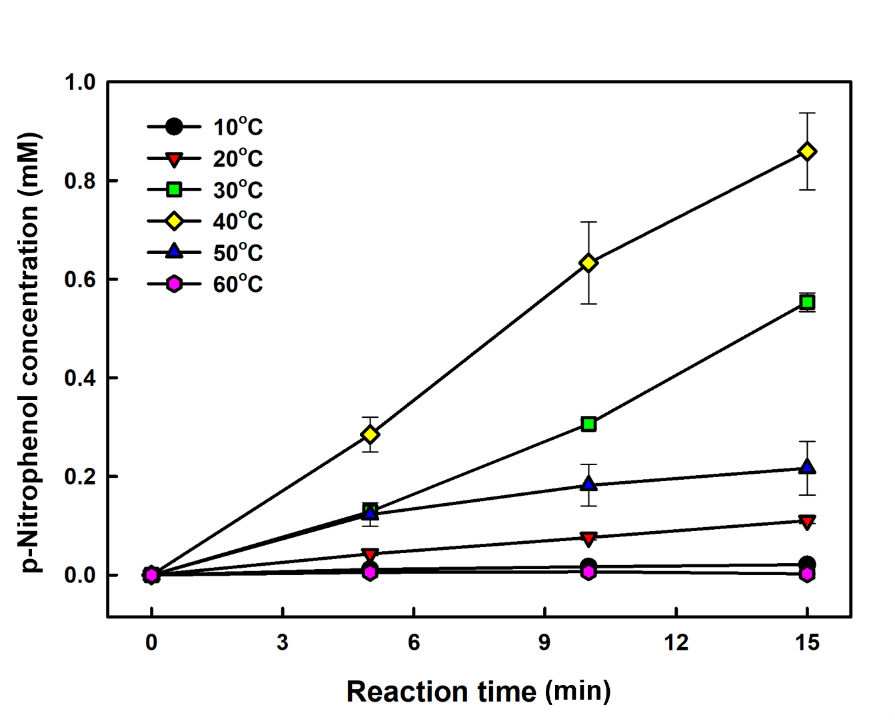


**Fig. S1. *p*-NPP assay results of FGC by temperature variation.** A total of 2 mL of reaction systems containing final 1 mM of *p*-NPP with surfactants were used with 50 mM Tris-HCl (pH 9.0) and 200 µL sampled at each measurement time. Shaking: magnetic stirring with 300 rpm.


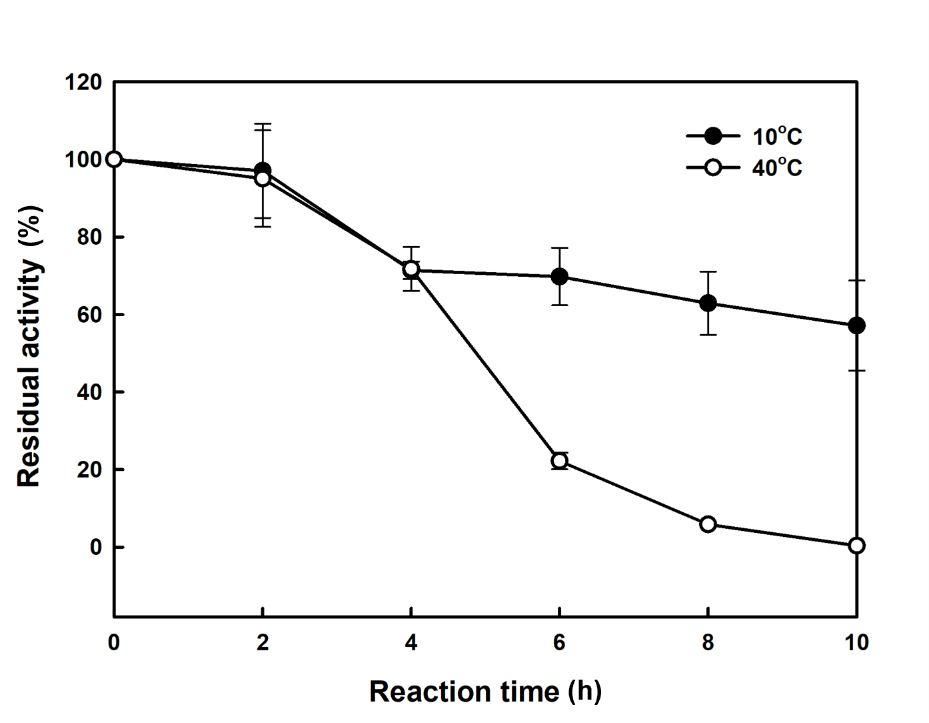


**Fig. S2. *p*-NPP activities of water phase in water–isooctane biphasic reaction system with FGC.** Isooctane without lipase substrate was used as organic phase. Residual activity was measured by *p*-NPP assay.


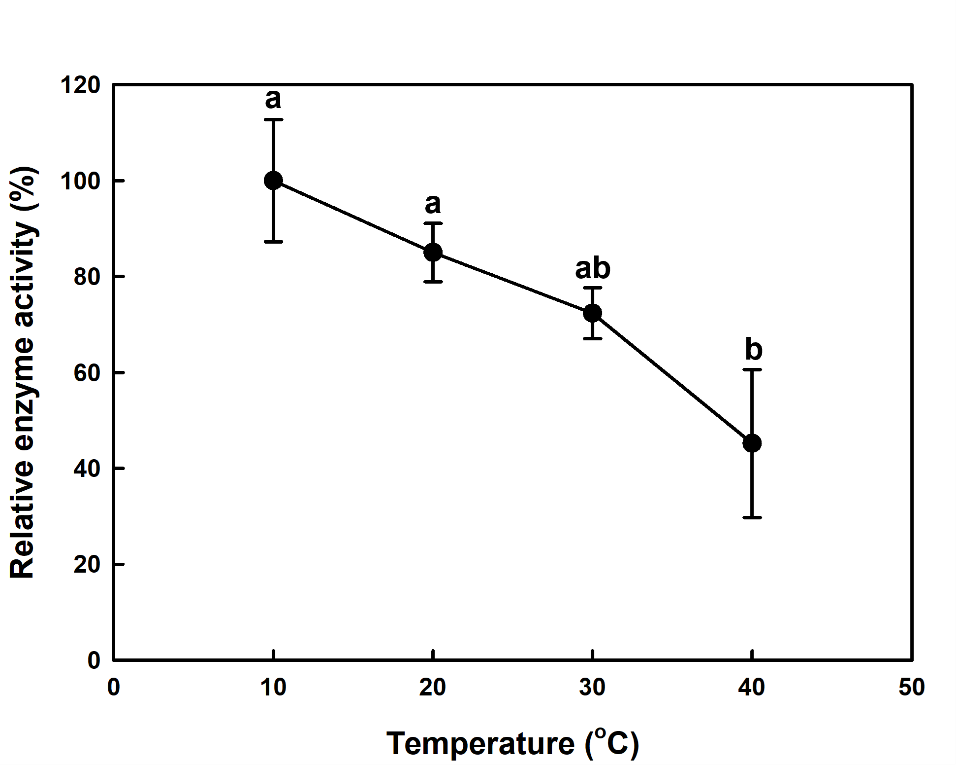


**Fig. S3. Lipase assay results for *p*-NPP of FGC in water–isooctane biphasic system by temperature variation.** As a substrate solution, 1 mM of triolein in isooctane was used and 50 mM Tris-HCl (pH 8.0) buffer was used as an enzyme solution.


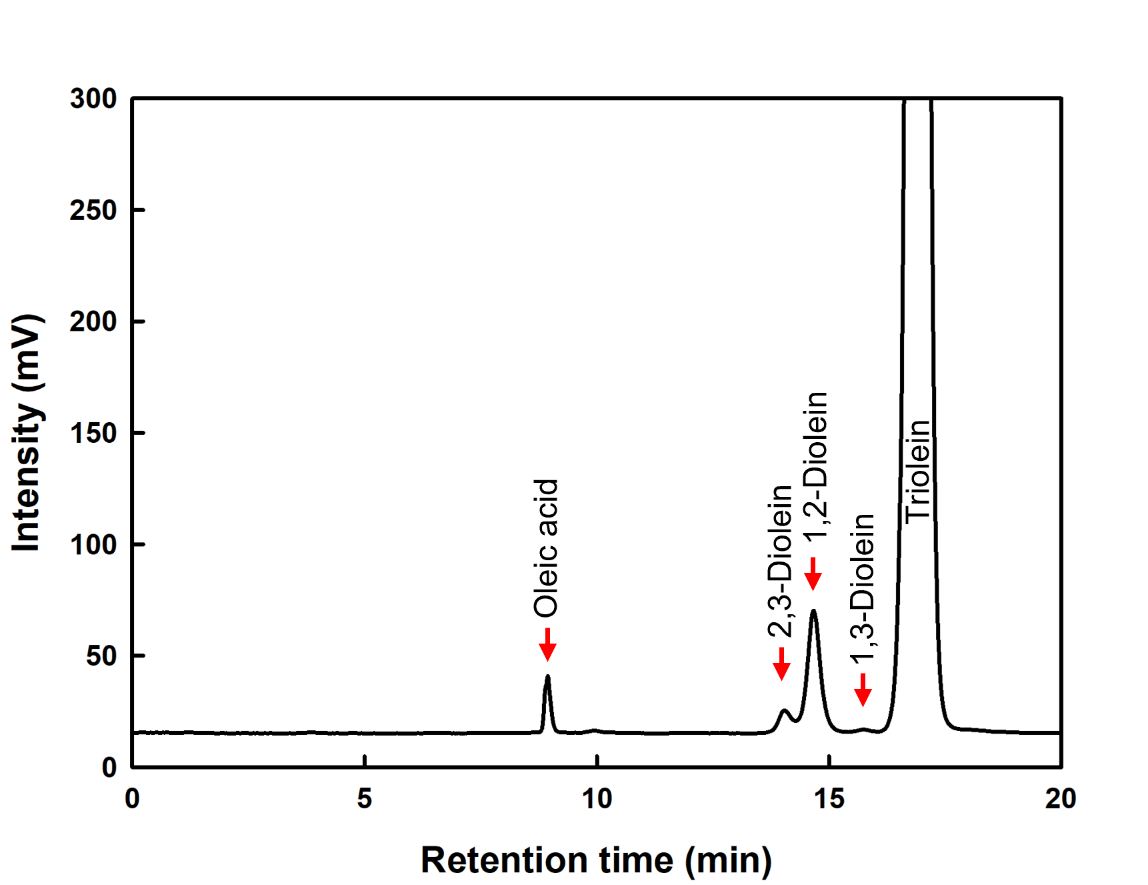


**Fig. S4. Chromatogram of HPLC analytes in hydrolysis at around 5% hydrolysis rate of triolein with FGC.** Concentrations of *sn*-1,3-diolein were below detection limit around all reaction time.
